# Yang-Mills structure for electron–phonon interactions in vanadium dioxide

**DOI:** 10.1038/s41598-020-68958-4

**Published:** 2020-07-27

**Authors:** Jamie M. Booth, Salvy P. Russo

**Affiliations:** 1 ARC Centre of Excellence in Exciton Science, RMIT University, Melbourne, VIC 3001 Australia; 2 Theoretical Chemical and Quantum Physics, RMIT University, Melbourne, VIC 3001 Australia

**Keywords:** Condensed-matter physics, Electronic properties and materials, Magnetic properties and materials, Semiconductors, Spintronics, Superconducting properties and materials

## Abstract

This work presents a method of grouping the electron spinors and the phonon modes of metal oxide crystals such as vanadium dioxide into an SU(2) gauge theory. The gauge “charge” is the electron spin, which is assumed to couple to the transverse acoustic phonons on the basis of spin ordering phenomena in ${\text{M}}_{{1}}$- and ${\text{M}}_{{2}}$-${\text{VO}}_{{2}}$, while the longitudinal mode is neutral. A generalization of the Peierls Mechanism is presented based on the discrete gauge invariance of crystals and the corresponding Ward-Takahashi identity. The introduction of a band index results in violation of this discrete Ward-Takahashi identity for interband transitions, resulting in scattering from the longitudinal component. Thus both the spinors and the bosons acquire mass and an electronic band gap and optical phonon modes result: a symmetry-breaking metal-insulator transition, which can manifest concurrent spin-ordering.

## Introduction

There currently exist a number of seemingly intractable problems in Condensed Matter physics (by intractable it is meant that some decades have passed since they were first identified without a solution being found). Mechanisms of metal-insulator transitions in metal oxides^1,2^ and high temperature superconductivity in the cuprates and pnictides are two examples^3,4^. In addition, the cooperative interplay of magnetism and lattice distortions has also been emphasized in layered transition-metal dichalcogenides^5–7^, but a complete and convenient mathematical description is yet to be published.

The metal-insulator transitions of polymorphs of vanadium oxide are one example of an area of research which hs defied complete mathematical description. The most famous example of these, vanadium dioxide^8^, undergoes a structural phase transition which coincides (roughly, there is some nuance here) with a metal-insulator transition at approximately 340 K. The high temperature structure is tetragonal (${\text{P4}}_{{2}}$/mnm, 136) which is metallic, while on the other side of ${\text{T}}_{{c}}$ the crystal adopts a monoclinic form (${\text{P2}}_{{1}}$/c, 14)^9,10^. The structural distortions occurring are in essence a pairing of the vanadium atoms along the tetragonal ${\mathbf{c}}$-axis, along with an antiferroelectric distortion orthogonal to this pairing, which has components in the tetragonal ${\mathbf{a}}$- and ${\mathbf{b}}$-axes.

The controversy surrounding ${\text{VO}}_{{2}}$ stems from the fact that the pairing of the vanadium atoms when going from the tetragonal to the monoclinic structure resulting in a gap opening seems to be an archetypal “Peierls pairing”^11^, which perturbation theory suggests would open a band gap. However, there is strong experimental evidence for an excitation gap due to strong electron correlations: the Mott-Hubbard mechanism^12^. While a band theoretical mechanism was proposed by Goodenough some decades ago^9^. Mott and Zylberstein^13^ argued in favour of the excitation gap resulting from localization due to strong electron-electron interactions on the basis of magnetic susceptibility measurements not long afterward. The transfer of spectral weight across the transition identified by Qazilbash et al.^14^ is also typical of correlation driven metal-insulator transitions. The reader is directed to Liu et al.^15^ for a thorough review.

There is both experimental and theoretical support for the involvement of phonons in the transition, with diffuse X-ray scattering^16^ and more recently inelastic X-ray scattering^17^ suggesting softening at the tetragonal R-point occurring, which aligns with the symmetry-breaking of the transformation. However, the question of whether the lattice softening, or electron correlations drive the transition is now recognized as misleading. In reality both are intertwined, which has been termed “correlation assisted Peierls” process or “Peierls-Mott” insulating behaviour by some^18,19^.

**Figure 1:**
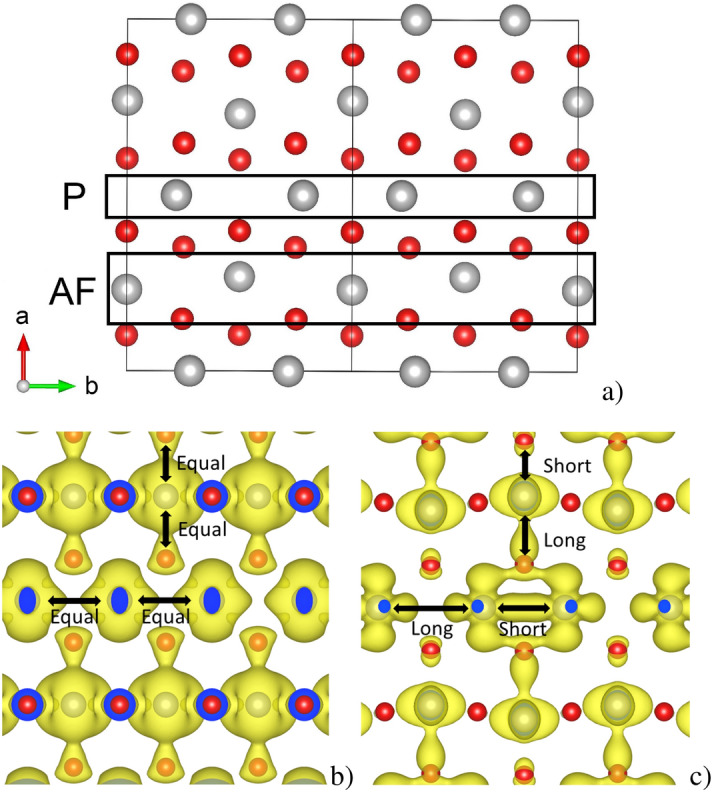
(**a**) Crystal structure of the ${\text{M}}_{{2}}$ form of vanadium dioxide viewed down the monoclinic c-axis, where “P” denotes the paired chain, while “AF” denotes the antiferroelectrically distorted chain, (**b**) Isosurface of the charge density of the tetragonal VO$_{2}$ structure, and (**c**) Isosurface of the charge density of the ${\text{M}}_{{2}} \, {\text{VO}}_{{2}}$ structure. Image produced using VESTA^24^ 3.1.5 (https://jp-minerals.org/vesta/en/).

Of interest in this work is the appearance of another insulating form of ${\text{VO}}_{{2}}$, the ${\text{M}}_{{2}}$ structure. The ${\text{M}}_{{2}}$ form of vanadium dioxide is also monoclinic^20^, and like the ${\text{M}}_{{1}}$ form it also undergoes a metal-insulator transition from the same rutile structure as ${\text{M}}_{{1}}$, albeit at a slightly elevated temperature^21^. However, this monoclinic structure has a particularly interesting feature, in that it is comprised of the same structural distortions as the ${\text{M}}_{{1}}$ form, but split across different vanadium chains. That is, in the ${\text{M}}_{{1}}$ form, all vanadium atoms pair up along the tetragonal c-axis, and at the same time undergo an antiferroelectric distortion, in which neighbouring metal ions displace in opposite directions along the long axis of the octahedron^20^. In the ${\text{M}}_{{2}}$ form these distortions occur on alternating chains, as Fig. 1a illustrates. The key piece of information is that the antiferroelectrically distorted chain also orders antiferromagnetically, while the paired chain does not^22^. Similar cooperation between charge and spin ordering resulting in Mott physics has also been recently reported in 1T-${\text{NbSe}}_{{2}}$^6^.

This suggests that the antiferromagnetic spin ordering is somehow related to the antiferroelectric distortion, while the pairing distortion has no effect on the spin. In this work we are interested determining how both the charge and spin-ordering seen in the metal-insulator transitions of ${\text{M}}_{{1}}$ and ${\text{M}}_{{2}} \, {\text{VO}}_{{2}}$ can arise out of a mixture of electron-electron and electron-phonon interactions. Specifically, if a phonon mode is described by a space- and time-varying polarization vector, how can these vector bosons interact with the spinor variables in a crystal to generate the required charge- and spin-ordering?

The formulation described here is concerned with symmetry-breaking, and in particular the formation of massive excitations from massless constituents (i.e. energy gaps), and is focused on electron-lattice interactions. It is found that an SU(2) gauge theory in which the transverse phonons are charged under the gauge group; that is they couple to the both electric charges of the electrons *and* their spins, while the longitudinal mode is neutral and induces only electric charge fluctuations, can describe crystal structure transformations which are accompanied by spin ordering. As a Yang-Mills theory it predicts a confined phase at low energies.

## Results

### Bosons and gauge “charge”

While performing GW calculations for a different study^23^, an anomalous rearrangement of charge density was observed in ${\text{M}}_{{2}} \, {\text{VO}}_{{2}}$. Figure 1b, c present GW calculations of the charge density of the *d*-band electrons in tetragonal ${\text{VO}}_{{2}}$, and ${\text{M}}_{{2}} \, {\text{VO}}_{{2}}$ respectively. The tetragonal structure exhibits charge density which is distributed equally between the vanadium and oxygen atoms, and does not accumulate in the inter-vanadium regions. However, the ${\text{M}}_{{2}}$ structure exhibits a very different charge ordering. From Fig. 1c it is apparent that as the tetragonal structure transforms to the ${\text{M}}_{{2}}$ form, one of the chains dimerizes, and charge density accumulates between the paired vanadium atoms, indicated by the “Short” label.

However, the antiferroelectrically distorted chain exhibits the opposite behaviour. The symmetric charge density of the tetragonal structure deforms such that more accumulates between the vanadium atom and one of the oxygens, however it accumulates in the *long* inter-atomic spacing. This is an effect of electrostatic repulsion of the apical oxygen atoms, outlining the potential well that the 3$d^{1}$ electrons bound to the metal atoms exhibit. The repulsion from the cage of oxygen atoms creates a well centred in the tetrahedron, and therefore any displacement of the metal ion along the Jahn-Teller axis of the octahedron will create an instantaneous field which breaks the inversion symmetry of the octahedral potential. The spin ordering observed in the antiferroelectric vanadium chain suggests this may be a significant effect, and in recent years the interplay between spin relaxation mechanisms and structural distortions has received attention in the context of the unusually long carrier lifetimes in organometal halide perovskites such as methylammonium lead iodide (MA-${\text{PbI}}_{{3}}$). In these systems this attention has focused on the role of spin state splitting due to the Rashba effect^25^. This effect arises from an inhomogeneous electric field which can alter orbital hybridization, leading to unusual spin relaxation mechanisms. Etienne et al.^26^ used a computational approach based on a combination of ab inito band structure and molecular dynamics calculations to show that a dynamic Rashba effect may be present in MA-${\text{PbI}}_{{3}}$, which was verified experimental by Niesner et al.^27^. A similar effect may be at play in vanadium dioxide systems, which exhibit strong electron-phonon interactions^16,17^, possibly as a result of strong electron-electron interactions^13,14,28^.

In the following work we take such a mechanism as an axiom: there is some effective interaction occurring, possibly a mixture of the Rashba effect, spin-obit coupling and strong exchange, which couples to certain phonon polarization vectors and flips electron spins, and explore its theoretical consequences. Thus, from the observed spin and charge ordering in ${\text{M}}_{{2}}$ vanadium dioxide and this axiomatic starting point, it seems that there may be *two* different effects of the metal atom motion on the localized electrons. There is a “Neutral” phonon, which affects the charge density, but not the spin. There are also “Charged” bosons, which can align the spin up- or down, which arise from polarization vectors orthogonal to the “Neutral” case. Such charged and uncharged bosons are functions of the coupling of the polarization vectors to the local environment of the metal atom, and thus would be expected to differ in materials in which this environment changes.

### Spinors and the Weyl equation

In problems such as metal-insulator transitions and superconductivity we are interested in the behavior of the electrons (and to some extent the lattice), and in particular the electrons on- or close to the Fermi surface, which act as metallic excitations before symmetry-breaking. For example in the cuprates these are the $d_{x^{2}-y^{2}}$ states, and in vanadium dioxide the vanadium $d^{1}$ states. It is therefore natural to concentrate solely on these degrees of freedom, and consider tight-binding wavefunctions for the electrons comprised of atomic-like orbitals:

1 $$\begin{aligned} {\psi}_{n{\mathbf{k}}}({\mathbf{r}}) = \sum _{j, {\mathbf{k}}} \phi _{j}({\mathbf{r}}-{\mathbf{R}})e^{i{\mathbf{k}}{\mathbf{R}}} \end{aligned}$$

where *n* is the band index, $\phi _{j}({\mathbf{r}})$ labels the atomic-like orbitals which are summed over to give the position state wavefunctions in each unit cell, ${\mathbf{R}}$ labels the set of lattice vectors which describe the translational symmetry of the lattice, and ${\mathbf{k}}$ is the wavevector which describes the spatial variation in the wavefunction amplitude.

As we are concentrating on the metallic states close to $E_{F}$, and assuming a 2-dimensional Fermi surface, the bands which form such a surface can be linearized at the Fermi wavevector: ${\mathbf{k}} = {\mathbf{k}}_{F}$. Shifting ${\mathbf{k}} \rightarrow {\mathbf{k-k_{F}}}$ then $E_{{\mathbf{k}}} = v_{F}\vert {\mathbf{k}}|$ and the states describe electrons and holes on or near the Fermi surface. However, unlike the Standard Model, the coordinate system of a crystal has a specific orientation, so no Poincaré group exists. Of course, there will be a discrete rotational and translational symmetry of the crystal given by its space group, but in general this will not be of much use to us, as it is not a Lie Group and therefore the considerable machinery of Poincaré invariance cannot be applied.

Therefore, it is important to the discussion below that momentum states in crystals are, in general, not related by a simple transformation. We can state that apart from rotations and translations of the space group, momenta are related by *scattering* processes, not symmetry transformations. Of course, momenta which are related by scale transformations: ${\mathbf{k}}\rightarrow \alpha {\mathbf{k}}$, are related. This may seem extremely restrictive, but it is actually a considerable simplification. We can treat each radial *direction* in momentum space separately, and sum over them to give the total result. For effects restricted to the Fermi surface this is simply equivalent summing over each point on the Fermi surface.

There is one symmetry operation which will be of considerable use; which is that for tetragonal (metallic) vanadium dioxide an inversion centre exists. Therefore there *is* a symmetry operation relating stationary momentum states ${\mathbf{k}}$ and $-{\mathbf{k}}$. It is straightforward to prove that these states satisfy the Weyl equation in (3+1) dimensions, as for each individual pair we can rotate the coordinate axes such that $k = (\frac{E}{v_{F}},0,0,k)$ giving the Weyl equation:

2 $$\begin{aligned} i\gamma ^{\mu }\partial _{\mu }{\psi}_{{\mathbf{a}}} = \begin{pmatrix} 0&{}0&{}\frac{E}{v_{F}}-k&{}0\\ 0&{}0&{}0&{}\frac{E}{v_{F}}+k\\ \frac{E}{v_{F}}+k&{}0&{}0&{}0\\ 0&{}\frac{E}{v_{F}}-k&{}0&{}0 \end{pmatrix}\, \begin{pmatrix} \psi ^{1}_{L}\\ \psi ^{2}_{L}\\ \psi ^{1}_{R}\\ \psi ^{2}_{R} \end{pmatrix}=0 \end{aligned}$$

using

3 $$\begin{aligned} {\psi}_{{\mathbf{a}}}=\begin{pmatrix} {\psi}_{L}\\ {\psi}_{R} \end{pmatrix} \end{aligned}$$

where the left-handed (*L*) and right-handed (*R*) states correspond to the two opposite helicity solutions (where the helicity operator is ${\sigma }^{i}p_{i}$, i.e. the Pauli matrices dotted into the momentum components) occurring for the up- and down spin degrees of freedom of the electrons. It must be stressed that this equation holds only for metallic systems, i.e. when electronic excitations can be achieved at arbitrarily small energies. Thus the dispersion relation $E = v_{F}|k |$ gives a Weyl, and not a Dirac, equation as at zero $|k |$, i.e. at the Fermi surface the excitation energy is zero. Thus as the Fermi surface there is no energy gap, or “mass”. However, while this can be done for each *pair* of 3-momenta, ${\mathbf{k}}$ and $-{\mathbf{k}}$, the lattice structure does not have Poincaré invariance. Therefore, to be able to compare different momentum states, we need a way of satisfying $E=v_{F}|k|$ and also Eq. (2). The simplest method of achieving this is to allow complex momenta, and indeed this is also the manner in which the violation of Poincaré invariance is handled in modern amplitude methods such as BCFW recursion^29^. Of course, for a complete theory we need solutions for the left- and right-handed states, but first we need to determine how the lattice can influence how they vary from point-to-point across the lattice.

### Yang-Mills theory

A *U*(1) gauge theory introduces interactions using a covariant derivative which couples the spinor and vector fields:

4 $$\begin{aligned} \gamma^{\mu}D_{\mu }\psi = \gamma^{\mu}\big (\partial _{\mu } + ig{\hat{A}}_{\mu }\big )\psi \end{aligned}$$

where *g* is the coupling strength. This generates a Lagrangian which is invariant under local *U*(1) transformations, and describes electromagnetism, and if Lorentz invariance is relaxed, i.e. the vector boson ${\hat{A}}_{\mu }$ becomes a three vector: ${\hat{A}}_{i}$ where *i* indexes the dimensions of space, it can describe electron-phonon interactions. A Yang-Mills theory promotes this to *SU*(*N*) transformations, which can be represented by a basis of $N\times N$ matrices, or generators:

5 $$\begin{aligned} \gamma^{\mu}\big (\partial _{\mu } + ig{\hat{A}}_{\mu }\big )\psi \rightarrow \gamma^{\mu}\big (\partial _{\mu } + ig{\hat{T}}^{a}{\hat{A}_{\mu}}^{a}\big )\psi \end{aligned}$$

where the ${\hat{T}}^{a}$ are the basis matrices parametrizing the space of *SU*(*N*) transformations (the generators), and the index *a* runs from $1 \rightarrow N$. There are now $N^{2}-1$ types of boson, not one, and as they are matrix valued they do not necessarily commute (in mathematical parlance the gauge group is non-Abelian). Since these generators are $N \times N$, they must act on column matrices of *N* 4-component spinors. In the *SU*(2) theory considered in this work *N* is two, and therefore the spinors the vector field acts on are double-stacked 4 component spinors, and there are three types of boson. A generator which is diagonal will not exchange the upper and lower spinors in an *SU*(2) interaction and thus this generator is said to generate “neutral” bosons. Generators with only off-diagonal components will exchange the upper and lower spinors, and these are the “charged” bosons. In this work, when a bososn is referred to as charged, by this it is meant that it contains only off-diagonal elements in its generator. The reason for such nomenclature is that in Yang-Mills theory the *N* stacked spinors carry *N* different generalized “charges”. In this work the “charged” gauge bosons couple to the electron spin, and therefore the gauge “charge” is spin.

For the vector fields (phonons) we can make the assumption that the solution to the field equation will be of the form of a polarization vector varying in time and space^30^:

6 $$\begin{aligned} W^{a}_{\mu }(x)\sim \sum _{p}\epsilon _{\mu }(p)e^{ipx} \end{aligned}$$

where $\epsilon _{\mu }(p)$ is the polarization vector for each momentum state *p*. Each boson can be quantized as per:

7 $$\begin{aligned} {\hat{W}}_{\mu }^{a}(x) = \int \frac{d^{3}p}{{2\pi }^\frac{3}{2}2 E_{{\mathbf{p}}}^{\frac{1}{2}}}\sum _{\lambda }\big [{\hat{a}} \epsilon ^{\lambda }_{\mu }(p)e^{ip_{\mu }x^{\mu }} + {\hat{a}}^{\dagger }\epsilon ^{*\lambda }_{\mu }(p)e^{-ip_{\mu }x^{\mu }}\big ] \end{aligned}$$

where $\mu$ is a spacetime index running from $0 \rightarrow 3$, *a* is an index running from $1\rightarrow 3$ indicating the boson “colour”, $x = (x^{0},x^{i}) = (t,{\mathbf{x}})$, and $\epsilon _{\mu }^{\lambda }(p)$ is the polarization vector as per Eq. (6) expressed with basis $\lambda$, and we approximate the Brillouin Zone sum by an integral. The factor of $\sqrt{2E_{{\mathbf{p}}}}^{-1}$ ensures that the field operator equal time commutation relation is obeyed:

8 $$\begin{aligned} \big [\dot{W_{u}}({\mathbf{x}},t),W_{\mu }({\mathbf{x}}^{\prime },t)\big ] = -i\delta ({\mathbf{x}}-{\mathbf{x}}^{\prime }) \end{aligned}$$

Of note: as Lorentz invariance is not present, the polarization vectors $\epsilon _{\mu }$ are 3-vectors, not 4-vectors, and thus so are the vector bosons. However, the covariant derivative and the field strength tensors for the bosons do contain time-derivatives and therefore some indices run over three values, and some over four. We have decided that the easiest way to approach this is to keep using $\mu$ as a label running from $0 \rightarrow 3$, and simply set the time component $\epsilon _{0}$ of the polarization vectors to zero when necessary (such as in the discussion of the Ward-Takahashi identity below).

These bosons can be used to define an interaction vertex in which the charge and spin density of the electrons couple to the vibrational modes, however to do this it is necessary to determine how to arrange the electron states into spinors which interact with these bosons such that the correct behaviour emerges.

Fortunately the Nambu spinors provide one such arrangement, although this is not obvious at face value. This gives as possibilities:

9 $$\begin{aligned} {\hat{\psi }}_{\mathbf{a,b}} = \begin{pmatrix} {\hat{c}}_{{\mathbf{k}}\uparrow }\\ {\hat{c}}^{\dagger }_{\mathbf{-k}\downarrow } \end{pmatrix}, \begin{pmatrix} {\hat{c}}_{\mathbf{-k}\downarrow }\\ {\hat{c}}^{\dagger }_{{\mathbf{k}}\uparrow } \end{pmatrix}, \begin{pmatrix} {\hat{c}}^{\dagger }_{{\mathbf{k}}\downarrow }\\ {\hat{c}}_{\mathbf{-k}\uparrow } \end{pmatrix}, \begin{pmatrix} {\hat{c}}^{\dagger }_{\mathbf{-k}\uparrow }\\ {\hat{c}}_{{\mathbf{k}}\downarrow } \end{pmatrix} \end{aligned}$$

where ${\mathbf{a}}$ and ${\mathbf{b}}$ are, for the moment, simply labels for the spinors, and we have used the 3-vectors to label the momenta to express the helicities more clearly. Parametrizing the SU(2) interaction vertex using the Pauli matrices as generators, and using the notation:

10 $$\begin{aligned} \sigma _{a}\cdot {\hat{W}}^{a}_{\mu }\rightarrow {\hat{W}}^{a}_{\mu } \end{aligned}$$

where $a = 1, 2, 3$ labels the generator, $\mu$ is a spacetime index and the $\sigma _{a}$ are:

11 $$\begin{aligned} \sigma _{1} = \begin{pmatrix} 0&{}1\\ 1&{}0 \end{pmatrix}; \sigma _{2} = \begin{pmatrix} 0&{}-i\\ i&{}0 \end{pmatrix}; \sigma _{3} = \begin{pmatrix} 1&{}0\\ 0&{}-1 \end{pmatrix} \end{aligned}$$

therefore we have:

12 $$\begin{aligned} {\hat{W}}^{a}_{\mu }= \begin{bmatrix} {\hat{W}}^{3}_{\mu }&{}{\hat{W}}^{1}_{\mu }-i{\hat{W}}^{2}_{\mu } \\ {\hat{W}}^{1}_{\mu }+i{\hat{W}}^{2}_{\mu }&{}-{\hat{W}}^{3}_{\mu } \end{bmatrix} \end{aligned}$$

From this vertex we see that the ${\hat{W}}^{1}$ and ${\hat{W}}^{2}$ bosons are off-diagonal are therefore the “charged bosons”, while the ${\hat{W}}^{3}$ bosons is diagonal and therefore “neutral”. We then expect electron-phonon interactions to be of the form:

13 $$\begin{aligned} g_{a}{\bar{\psi }}\gamma ^{\mu }{\hat{W}}^{a}_{\mu }\psi =g_{(1,2,3)} \begin{pmatrix} {\bar{\psi }}_{\mathbf{a}}^{\prime },{\bar{\psi }}_{{\mathbf{b}}^{\prime }} \end{pmatrix}\gamma ^{\mu } \begin{pmatrix} {\hat{W}}^{3}_{\mu }&{}{\hat{W}}^{1}_{\mu }-i{\hat{W}}^{2}_{\mu }\\ {\hat{W}}^{1}_{\mu }+i{\hat{W}}^{2}_{\mu }&{}-{\hat{W}}^{3}_{\mu } \end{pmatrix}\, \begin{pmatrix} {\psi}_{{\mathbf{a}}} \\ {\psi}_{{\mathbf{b}}} \end{pmatrix} \end{aligned}$$

where the gamma matrices are expressed (in the chiral basis) in two-component form as:

14 $$\begin{aligned} \gamma ^{0} = \begin{pmatrix} 0&{} {1}\\  {1}&{}0 \end{pmatrix}, \gamma ^{i} = \begin{pmatrix} 0&{}\sigma ^{i}\\ -\sigma ^{i}&{}0 \end{pmatrix} \end{aligned}$$

and thus

15 $$\begin{aligned} {\bar{\psi }} = \psi ^{\dagger }\gamma ^{0} = ({\bar{\psi }}_{{\mathbf{a}}},{\bar{\psi }}_{{\mathbf{b}}}) = (\psi ^{\dagger }_{{\mathbf{a}}}\gamma ^{0},\psi ^{\dagger }_{{\mathbf{b}}}\gamma ^{0}) \end{aligned}$$

### Diagonal interactions

Setting $F^{1}_{\mu \nu }=F^{2}_{\mu \nu } = 0$ for the field strength tensors gives:

16 $$\begin{aligned} \begin{pmatrix} {\bar{\psi }}_{\mathbf{a}}^{\prime },{\bar{\psi }}_{{\mathbf{b}}^{\prime }} \end{pmatrix}g_{3}\gamma ^{\mu } \begin{pmatrix} {\hat{W}}^{3}_{\mu }&{}0\\ 0&{}-{\hat{W}}^{3}_{\mu } \end{pmatrix}\, \begin{pmatrix} {\psi}_{{\mathbf{a}}}\\ {\psi}_{{\mathbf{b}}} \end{pmatrix}={\bar{\psi }}_{\mathbf{a}}^{\prime }g_{3} \gamma ^{\mu }{\hat{W}}^{3}_{\mu }{\psi}_{{\mathbf{a}}}- {\bar{\psi }}_{\mathbf{b}}^{\prime }g_{3}\gamma ^{\mu }{\hat{W}}^{3}_{\mu }{\psi}_{{\mathbf{b}}} \end{aligned}$$

where $i = 1, 2, 3$. These are the familiar matrix elements of a standard Abelian gauge theory, which represent the traditional electron-phonon interaction involved in for example the BCS theory of superconductivity, with the exception that the Yang-Mills field strength tensor $F^{3}_{\mu \nu }$ contains a quadratic term which gives self-interactions. In the language of differential forms: $F^{3}_{\mu \nu } = dF^{3}_{\mu } + F^{1}_{\mu }\wedge F^{2}_{\nu }$. For conventional electron-phonon interactions, for example in monovalent metals, the assumption that the oxygen ligands create fields which influence the spin dynamics is not valid and $F^{1}_{\mu \nu }=F^{2}_{\mu \nu } \sim 0$ and the quadratic term vanishes, giving the standard Abelian Field Strength Tensor. In this respect, the ${\hat{W}}^{3}_{\mu }$ boson is like the neutral boson of the weak interaction.

However, despite this apparent simplicity there is an additional subtlety to do with the polarization vectors and their actions on the atoms. Remembering the ansatz:

17 $$\begin{aligned} {\hat{W}}^{a}_{\mu }(x)\sim \sum _{p}{\hat{a}}^{\dagger }_{p}\epsilon _{\mu }(p)e^{-ipx} + H.c. \end{aligned}$$

we see that dotting this into the $\sigma _{3}$ matrix will give:

18 $$\begin{aligned} \begin{pmatrix} {\hat{W}}^{3}_{\mu }&{}0\\ 0&{}-{\hat{W}}^{3}_{\mu } \end{pmatrix}\rightarrow \sum _{p} \begin{pmatrix} \epsilon _{\mu }(p)e^{ipx}&{}0\\ 0&{}-\epsilon _{\mu }(p)e^{ipx} \end{pmatrix} \end{aligned}$$

therefore the negative sign on the lower operator reverses the polarization vector, and thus this vertex cannot describe interactions at the same unit in the crystal, as the atom(s) would need to move in two directions at once. However, they can act on *neighbouring* unit cells, which would give a *pairing* of atoms in neighbouring unit cells for a polarization vector with a component along the axis connecting the atoms.

Thus for two neighbouring atoms *i* and *j* in a crystal we write the interactions of the ${\hat{W}}^{3}_{\mu }$ with the spinors as:

19 $$\begin{aligned} g_{3}({\bar{\psi }}^{\prime }_{{\mathbf{a}}}(x_{i}), {\bar{\psi }}^{\prime }_{{\mathbf{b}}}(x_{j}))\gamma ^{\mu } \begin{pmatrix} {\hat{W}}^{3}_{\mu }(x_{i})&{}0 \\ 0&{}-{\hat{W}}^{3}_{\mu }(x_{j}) \end{pmatrix} \,\begin{pmatrix} {\psi}_{{\mathbf{a}}}(x_{i})\\ {\psi}_{{\mathbf{b}}}(x_{j}) \end{pmatrix} \end{aligned}$$

Thus this SU(2) Yang-Mills type vertex which contains pairing motions on neighbouring sites corresponds to momentum states in which $\frac{\pi }{2{\mathbf{a}}} \leq {\mathbf{k}} \leq \frac{\pi }{{\mathbf{a}}}$, and in a symmetry-broken state will describe *optical* phonons.

### Off-diagonal terms

This formulation in which the diagonal boson sit on the links between neighbouring unit cells has significant consequences for the off-diagonal bosons, however we will see that this *also* turns out to be necessary; the Yang-Mills vertex as formulated here sits naturally on the boundary between two unit cells.

In order to contain gauge “charge” coupling the off-diagonal terms contain spin raising and lowering operators. To see how these arise, we set ${\hat{W}}^{3}_{\mu }=0$ for clarity and expand the interaction for $\mu = 1,2$ to get:

20 $$\begin{aligned}({\bar{\psi }}_{\mathbf{a}}^{\prime },{\bar{\psi }}_{{\mathbf{b}}^{\prime }}) g_{(1,2)}\gamma ^{\mu } & \begin{pmatrix} 0&{}{\hat{W}}^{1}_{\mu }-i{\hat{W}}^{2}_{\mu }\\ {\hat{W}}^{1}_{\mu }+i{\hat{W}}^{2}_{\mu }&{}0 \end{pmatrix}\,\begin{pmatrix} {\psi}_{{\mathbf{a}}}\\ {\psi}_{{\mathbf{b}}} \end{pmatrix} \\&\quad = ({\bar{\psi }}_{\mathbf{a}}^{\prime },{\bar{\psi }}_{{\mathbf{b}}^{\prime }}) g_{(1,2)} \begin{pmatrix} 0&{}\gamma ^{1}\\ \gamma ^{1}&{}0 \end{pmatrix}\,\begin{pmatrix} 0&{}{\hat{W}}^{1}_{1}-i{\hat{W}}^{2}_{1}\\ {\hat{W}}^{1}_{1}+i{\hat{W}}^{2}_{1}&{}0 \end{pmatrix}\,\begin{pmatrix} {\psi}_{{\mathbf{a}}}\\ {\psi}_{{\mathbf{b}}} \end{pmatrix} \\&\qquad +\,({\bar{\psi }}_{\mathbf{a}}^{\prime },{\bar{\psi }}_{{\mathbf{b}}^{\prime }}) g_{(1,2)} \begin{pmatrix} 0&{}\gamma ^{2}\\ \gamma ^{2}&{}0 \end{pmatrix}\,\begin{pmatrix} 0&{}{\hat{W}}^{1}_{2}-i{\hat{W}}^{2}_{2}\\ {\hat{W}}^{1}_{2}+i{\hat{W}}^{2}_{2}&{}0 \end{pmatrix} \, \begin{pmatrix} {\psi}_{{\mathbf{a}}}\\ {\psi}_{{\mathbf{b}}} \end{pmatrix} \end{aligned}$$

Setting $g_{1}{\hat{W}}^{1}_{1} = g_{2}{\hat{W}}^{2}_{2}$ to illustrate this most clearly we get a term:

21 $$\begin{aligned} \begin{pmatrix} {\bar{\psi }}_{\mathbf{a}}^{\prime },{\bar{\psi }}_{{\mathbf{b}}^{\prime }} \end{pmatrix}\, \begin{pmatrix} 0&{}g_{1}{\hat{W}}^{1}_{1}(\gamma ^{1}-i\gamma ^{2})\\ g_{1}{\hat{W}}^{1}_{1}(\gamma ^{1}+i\gamma ^{2})&{}0 \end{pmatrix}\, \begin{pmatrix} {\psi}_{{\mathbf{a}}}\\ {\psi}_{{\mathbf{b}}} \end{pmatrix} \end{aligned}$$

If both ${\psi}_{{\mathbf{a}}}$ and ${\psi}_{{\mathbf{b}}}$ are in eigenstates of $S_{Z}$, and remembering:

22 $$\begin{aligned} \gamma ^{i} = \begin{pmatrix} 0&{}\sigma ^{i}\\ -\sigma ^{i}&{}0 \end{pmatrix} \end{aligned}$$

this gives the familiar spin raising and lowering operators, $S^{+}=\sigma ^{1}+i\sigma ^{2}$, and $S^{-}=\sigma ^{1}-i\sigma ^{2}$:

23 $$\begin{aligned} {\bar{\psi }}_{\mathbf{a}}^{\prime }g_{1}{\hat{W}}^{1}_{1} \begin{pmatrix} 0&{}{\hat{S}}^{-}\\ -{\hat{S}}^{-}&{}0 \end{pmatrix} {\psi}_{{\mathbf{b}}}+{\bar{\psi }}_{\mathbf{b}}^{\prime }g_{1} {\hat{W}}^{1}_{1} \begin{pmatrix} 0&{}{\hat{S}}^{+}\\ -{\hat{S}}^{+}&{}0 \end{pmatrix}{\psi}_{{\mathbf{a}}} \end{aligned}$$

with the negative sign in the $\gamma ^{i}$ accounting for the opposite helicities of the two-component spinors in each four-component spinor such that the Weyl equation for each is satisfied. This indicates that the use of the Pauli matrices as generators in the SU(2) theory endows the formalism with spin raising and lowering operators, allowing spin fluctuations to result from phonons acting on the electrons.

### Spin ordering

While it is straightforward to define the spin raising and lowering operators as per Eq. (23), as stated above there is also an additional subtlety to their implementation. If the action of the boson on the spin of an electron is determined by its polarization vector, and the phonons are assumed to be comprised of normal modes, the spin operators themselves will oscillate between raising and lowering as a function of time due to the oscillatory behaviour of the normal modes. For example at spacetime point *x* (i.e. a particular unit cell at a particular time) we might have as per Eq. (21):

24 $$\begin{aligned} \begin{pmatrix} {\bar{\psi }}_{\mathbf{a}}^{\prime }(x),{\bar{\psi }}_{{\mathbf{b}}^{\prime }}(x) \end{pmatrix}\, \begin{pmatrix} 0&{}g_{1}{\hat{W}}^{1}_{1}(\gamma ^{1}-i\gamma ^{2})(x)\\ g_{1}{\hat{W}}^{1}_{1}(\gamma ^{1}+i\gamma ^{2})(x)&{}0 \end{pmatrix}\, \begin{pmatrix} {\psi}_{{\mathbf{a}}}(x)\\ {\psi}_{{\mathbf{b}}}(x) \end{pmatrix} \end{aligned}$$

However, the problem with this vertex is that if it is the direction of the motion of the metal atom within the cage of oxygen atoms in the octahedral cluster that interacts with the spin of a bound electron, then in this vertex the spin raising and lowering operators are acting at the same time. However, since this is at a single spacetime point, or single unit cell, the different spin operators are arising from the *same* atomic motion. This is unphysical.

However this can be resolved in the same manner as for the ${\hat{W}}^{3}$ mode, by applying the two bosons to neighbouring sites on the lattice:

25 $$\begin{aligned}\gamma ^{\mu }{\hat{W}}^{a}_{\mu }(x_{ij})\psi & = \gamma ^{\mu } \begin{pmatrix} 0&{}{\hat{W}}^{1}_{\mu }(x_{j})-i{\hat{W}}^{2}_{\mu }(x_{j})\\ {\hat{W}}^{1}_{\mu }(x_{i})+i{\hat{W}}^{2}_{\mu }(x_{i})e^{i\pi }&{}0 \end{pmatrix}\, \begin{pmatrix} {\psi}_{{\mathbf{a}}}(x_{i})\\ {\psi}_{{\mathbf{b}}}(x_{j}) \end{pmatrix} \\&\quad =\gamma ^{\mu } \begin{pmatrix} 0&{}{\hat{W}}^{1}_{\mu }(x_{j})-i{\hat{W}}^{2}_{\mu }(x_{j})\\ {\hat{W}}^{1}_{\mu }(x_{i})+i{\hat{W}}^{2}_{\mu }(x_{i})&{}0 \end{pmatrix}\, \begin{pmatrix} {\psi}_{{\mathbf{a}}}(x_{i})\\ {\psi}_{{\mathbf{b}}}(x_{j}) \end{pmatrix} \end{aligned}$$

where now $x_{ij}$ labels the link between atomic sites *i* and *j*.

**Figure 2:**
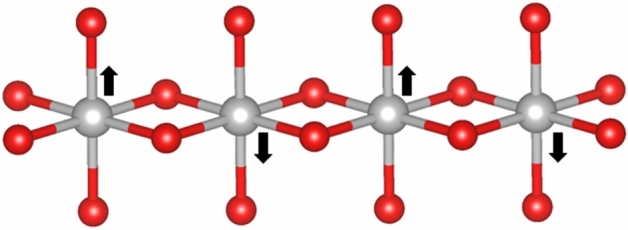
Antiferroelectric distortion of a chain of octahedrally coordinated metal atoms. Image produced using VESTA^24^ 3.1.5 (https://jp-minerals.org/vesta/en/).

To see how the interaction vertex can order spins antiferromagnetically along a chain of metal atoms, Fig. 2 illustrates a zone edge mode, in which neigbouring metal atoms experience identical magnetic fields oscillating in opposite directions due to the out-of-phase oscillations of the polarization vectors, thus the wavelength of such a mode is 2${\mathbf{a}}$, or twice the lattice spacing (i.e. $\mathbf{k=\frac{\pi }{{\mathbf{a}}}}$), if each octahedral cluster corresponds to one unit cell. This will order the spins antiferromagnetically, they will still oscillate from up-to-down, but 180 $^\circ$ out of phase. This is the type of “frozen phonon” seen to correspond to antiferromagnetic ordering in compounds such as ${\text{M}}_{{1}}$ and ${\text{M}}_{{2}} \, {\text{VO}}_{{2}}$ as a result of their structural phase transitions. If such ordering were to occur from coherent oscillations just above ${\text{T}}_{{c}}$, along with the symmetry-breaking described by the $\hat{W^{3}_{\mu }}$ mode above, at ${\text{T}}_{{c}}$ in the tetragonal structure of ${\text{VO}}_{{2}}$, then we might expect the formation of localized singlets on the paired vanadium atoms, along with the transition to monoclinic symmetry from the pairing and antiferroelectric distortion VEVs, echoing what is seen in experiment. Thus this formalism naturally contains the required operators to both pair neighbouring atoms on chains, and induce antiferromagnetic order, precisely the components required to describe the structural and electronic changes occurring in the metal-insulator transition of vanadium dioxide.

### Spinor grouping

This provides us with an easy way to determine how to group the Nambu spinors. If the interaction vertex is rewritten:

26 $$\begin{aligned} g_{a}{\bar{\psi }}\gamma ^{\mu }{\hat{T}}^{a}{\hat{W}}^{a}_{\mu }\psi = \begin{pmatrix} {\bar{\psi }}_{\mathbf{a}}^{\prime }(x_{i}),{\bar{\psi }}_{{\mathbf{b}}^{\prime }}(x_{j}) \end{pmatrix} g_{(+,-,3)}\gamma ^{\mu } \begin{pmatrix} {\hat{W}}^{3}_{\mu }(x_{i})&{}{\hat{W}}^{-}_{\mu }(x_{j})\\ {\hat{W}}^{+}_{\mu }(x_{i})&{}-{\hat{W}}^{3}_{\mu }(x_{j}) \end{pmatrix}\, \begin{pmatrix} {\psi}_{{\mathbf{a}}}(x_{i} \\ {\psi}_{{\mathbf{b}}}(x_{j}) \end{pmatrix} \end{aligned}$$

where $a = 1,2,3$, and is summed over, and ${\hat{W}}^{\pm }_{\mu } = {\hat{W}}^{1}_{\mu }\pm i{\hat{W}}^{2}_{\mu }$ then we group the spin down electrons and spin up holes into ${\psi}_{{\mathbf{a}}}$, and the spin up electrons and spin down holes into ${\psi}_{{\mathbf{b}}}$. Thus the ${\hat{W}}^{\pm }_{\mu }$ describe transformations between spinors which contain electrons of opposite momentum and spin if ${\hat{W}}^{1}_{\mu }$ and ${\hat{W}}^{2}_{\mu }$ are in phase (this can easily be generalized into arbitrary charge density relationships, which will be explored in the context of vanadium dioxide later). However, there are four Nambu spinors, and therefore there are two each of the ${\psi}_{{\mathbf{a}}}$ and ${\psi}_{{\mathbf{b}}}$. We can therefore group the spinors into flavours, and generations.

27 $$\begin{aligned} \begin{pmatrix} {\hat{c}}^{\dagger }_{{\mathbf{k}}\uparrow }\\ {\hat{c}}_{{\mathbf{-k}}\downarrow } \end{pmatrix} = {\text{up}},\quad \begin{pmatrix} {\hat{c}}^{\dagger }_{{\mathbf{-k}}\downarrow }\\ {\hat{c}}_{{\mathbf{k}}\uparrow } \end{pmatrix} = {\text{down}},\quad \begin{pmatrix} {\hat{c}}_{{\mathbf{k}}\downarrow }\\ {\hat{c}}^{\dagger }_{{\mathbf{-k}}\uparrow } \end{pmatrix} = {\text{top}},\quad \begin{pmatrix} {\hat{c}}_{\mathbf{-k}}\uparrow \\ {\hat{c}}^{\dagger }_{{\mathbf{k}}\downarrow } \end{pmatrix} = {\text{bottom}} \end{aligned}$$

**Figure 3:**
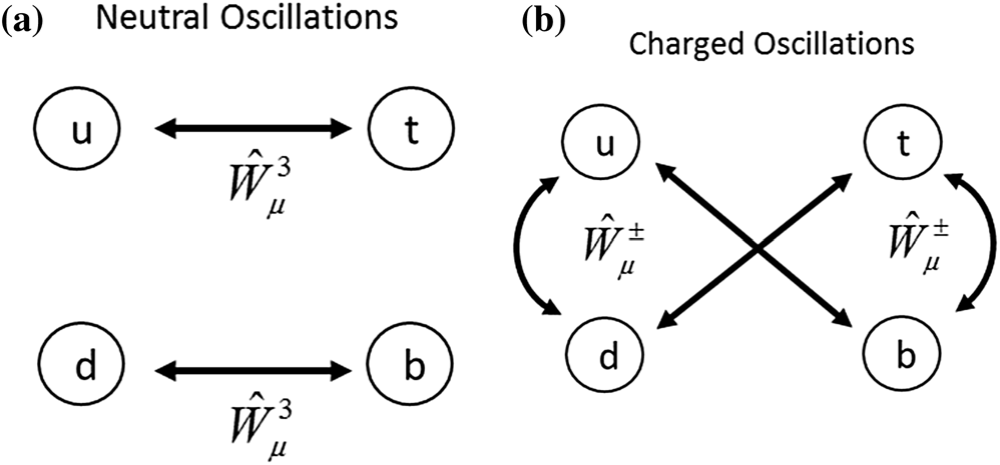
Schematic representation of the transformations enacted by the (**a**) Neutral boson ${\hat{W}}^{3}_{\mu }$ and (**b**) the Charged bosons ${\hat{W}}^{\pm }_{\mu }$.

The naming convention follows the spin of the holes in each Dirac spinor, which is done to preserve the commutation relations of the Pauli matrices. This is summarized in Table 1. This echoes the naming conventions for quarks in the Standard Model of Particle Physics, and deliberately so as it makes them somewhat easier to remember. Thus action of the phonons on the grouped Dirac spinors in all its gory detail becomes:

28 $$\begin{aligned} g_{(+,-,3)}\gamma ^{\mu }{\hat{W}}_{\mu }(x)\psi= & {} \begin{pmatrix} \gamma ^{\mu }{\hat{W}}^{3}_{\mu }(x_{1})&{} \gamma ^{\mu }{\hat{W}}^{-}_{\mu }(x_{2})\\ &{} \\ \gamma ^{\mu }{\hat{W}}^{+}_{\mu }(x_{1})&{}-\gamma ^{\mu }{\hat{W}}^{3}_{\mu }(x_{2}) \end{pmatrix}\, \begin{pmatrix} {\hat{c}}^{\dagger }_{{\mathbf{k}}\uparrow }\\ {\hat{c}}_{\mathbf{-k}}\downarrow \\ {\hat{c}}^{\dagger }_{\mathbf{-k}}\downarrow \\ {\hat{c}}_{{\mathbf{k}}\uparrow } \end{pmatrix} \\ g_{(+,-,3)}\gamma ^{\mu }{\hat{W}}_{\mu }(x)\psi= & {} \begin{pmatrix} \gamma ^{\mu }{\hat{W}}^{3}_{\mu }(x_{1})&{}\gamma ^{\mu }{\hat{W}}^{-}_{\mu }(x_{2})\\ &{} \\ \gamma ^{\mu }{\hat{W}}^{+}_{\mu }(x_{1})&{}-\gamma ^{\mu }{\hat{W}}^{3}_{\mu }(x_{2}) \end{pmatrix}\, \begin{pmatrix} {\hat{c}}_{{\mathbf{k}}\downarrow }\\ {\hat{c}}^{\dagger }_{\mathbf{-k}}\uparrow \\ {\hat{c}}_{\mathbf{-k}}\uparrow \\ {\hat{c}}^{\dagger }_{{\mathbf{k}}\downarrow } \end{pmatrix} \end{aligned}$$

where the positions of the spinors are inferred from the spacetime coordinates of the bosons to avoid cluttering the notation too much. Therefore ${\hat{W}}^{+}_{\mu }(x_{i})$ can scatter: a bottom to a top, a down to an up, and a bottom to an up (with zero wavevector) etc., and so on. A schematic of the transformations the bosons perform is presented in Fig. 3. Thus we see that a Yang-Mills vertex is a natural candidate for the charge and spin ordering seen in phase transitions of vanadium dioxide. The ${\hat{W}}^{+}_{\mu }(x_{i}), {\hat{W}}^{-}_{\mu }(x_{j})$ can order neighbouring spins antiferromagnetically, while the sign change for the lower ${\hat{W}}^{3}_{\mu }(x)$ boson reverses the direction of the polarization vector, and therefore results in neighbouring atoms pairing up, or “Peierls pairing.”

**Table 1:** Organization of the colors and generations of fermions in the SU(2) gauge theory.

Colour	Generation	
	1	2
**a**	Up	Top
**b**	Down	Bottom

Up and Top fermions (corresponding to the spinors in Eq. (27)) are color **a**, while the down and bottom spinors are color **b**.

### Mass generation

#### Spinor mass from neutral oscillations

Defining the chirality operator in the usual way using the matrix: $\gamma ^{5}=\gamma ^{0}\gamma ^{1}\gamma ^{2}\gamma ^{3}$ , we can redefine the spinors as per:

29 $$\begin{aligned} \frac{1}{2}(1-\gamma ^{5})\psi = \begin{pmatrix} {\psi}_{L}\\ 0 \end{pmatrix} = {\psi}_{L} \quad {\text{and}}\quad \frac{1}{2}(1+\gamma ^{5})\psi = \begin{pmatrix} 0\\ {\psi}_{R} \end{pmatrix} = {\psi}_{R} \end{aligned}$$

where:

30 $$\begin{aligned} \gamma ^{5} = \gamma ^{0}\gamma ^{1}\gamma ^{2}\gamma ^{3} = \begin{pmatrix} -1&{}0&{}0&{}0\\ 0&{}-1&{}0&{}0\\ 0&{}0&{}1&{}0\\ 0&{}0&{}0&{}1 \end{pmatrix} \end{aligned}$$

Thus:

31 $$\begin{aligned} \psi ^{1,2}_{{\mathbf{a}},{\mathbf{b}}} = {\psi}_{L}+{\psi}_{R} \end{aligned}$$

and identifying the upper and lower components of each 4-component spinor (i.e. the ${\psi}_{{\mathbf{a}}}$ and ${\psi}_{{\mathbf{b}}}$) as left- and right-handed chiral spinors (it is straightforward to prove these satisfy the Weyl equation in metallic systems), i.e.

32 $$\begin{aligned} \begin{pmatrix} {\hat{c}}^{\dagger }_{{\mathbf{k}}\uparrow }\\ {\hat{c}}_{\mathbf{-k}}\downarrow \end{pmatrix} = \begin{pmatrix} u_{L}\\ u_{R} \end{pmatrix}\quad {\text{and}}\quad \begin{pmatrix} {\hat{c}}_{{\mathbf{k}}\downarrow }\\ {\hat{c}}^{\dagger }_{\mathbf{-k}}\uparrow \end{pmatrix} = \begin{pmatrix} t_{L}\\ t_{R} \end{pmatrix} \end{aligned}$$

and

33 $$\begin{aligned} \begin{pmatrix} {\hat{c}}^{\dagger }_{\mathbf{-k}}\downarrow \\ {\hat{c}}_{{\mathbf{k}}\uparrow } \end{pmatrix} = \begin{pmatrix} d_{L}\\ d_{R} \end{pmatrix}\quad {\text{and}}\quad \begin{pmatrix} {\hat{c}}_{\mathbf{-k}}\uparrow \\ {\hat{c}}^{\dagger }_{{\mathbf{k}}\downarrow } \end{pmatrix} = \begin{pmatrix} b_{L}\\ b_{R} \end{pmatrix} \end{aligned}$$

we can see how the phonon field gaining a Vacuum Expectation Value (VEV) can result in massive spinors in the same manner as neutrino oscillations (i.e. the Rabi cycle). While all of the ${\hat{W}}^{a}_{\mu }$ contribute to lattice potential fluctuations, let us focus on ${\hat{W}}^{3}_{\mu }$ for clarity, and note that a longitudinal phonon with wavevector ± 2**k** will scatter $u_{L}\rightarrow t_{R}$, $u_{R}\rightarrow t_{L}$, $d_{L}\rightarrow b_{R}$ and $d_{R}\rightarrow b_{L}$ and vice versa, where ${\mathbf{k}}$ is the wavevector of the electron state. So, giving ${\hat{W}}^{3}_{\mu }$ a VEV ($\omega \rightarrow 0$, i.e. its time variation goes to zero) with wavevector $2{\mathbf{k}}$ we get:

34 $$\begin{aligned} {\hat{W}}^{3}_{\mu }(x)\rightarrow \langle {\hat{W}}^{3}_{0}\rangle +{\hat{W}}^{3}_{\mu }(x) \end{aligned}$$

where here the 0 subscript indicates a VEV and not a spacetime index. To maintain the spin ordering, i.e. to give neutral oscillations as per Fig. 3, there will be constraints on the polarization vector. Looking at the interaction of the boson with an incoming spinor such as $t_{L}$ (dropping the coupling constant and the outgoing spinor to see the interaction more clearly):

35 $$\begin{aligned} ig_{3}{\bar{\psi }}\gamma ^{\mu }{\hat{W}}^{3}_{\mu }\psi \rightarrow {\bar{\sigma }}^{\mu }\epsilon _{\mu }(p)e^{ipx} \begin{pmatrix} 0\\ 1 \end{pmatrix} e^{ikx}= & {} \begin{pmatrix} \epsilon _{0}-\epsilon _{3}&{}-(\epsilon _{1}-i\epsilon _{2}) \\ -(\epsilon _{1}+i\epsilon _{2})&{}\epsilon _{0}+\epsilon _{3} \end{pmatrix} \, \begin{pmatrix} 0\\ 1 \end{pmatrix} e^{i(p+k)x} \\= & {} \begin{pmatrix} -\epsilon _{1}+i\epsilon _{2}\\ \epsilon _{0}+\epsilon _{3} \end{pmatrix}e^{i(p+k)x} \end{aligned}$$

where ${\bar{\sigma }}=({1},-\sigma ^{i})$. By giving the ${\hat{W}}^{3}_{\mu }(x)$ field a VEV, and setting $\epsilon _{0} = 0$ to maintain the spin orientation we can have:

36 $$\begin{aligned} \epsilon _{1}=i\epsilon _{2},\quad {\text{or}}\quad \epsilon _{1}= \epsilon _{2}=0,\quad {\text{with}}\quad \epsilon _{3}=1 \end{aligned}$$

Choosing the easy path and defining the orientation of the polarization vector as being down the z-axis ($\epsilon _{\mu }(p)=(0,0,0,1)$) the full interaction vertex; $-g_{3}{\bar{\psi }}\gamma^{\mu }{\hat{W}}_{\mu }\psi$ gives:

37 $$\begin{aligned} g_{3}\langle W^{3}_{3}\rangle {\bar{u}}_{R}t_{L} + g_{3}\langle W^{3}_{3}\rangle {\bar{u}}_{L}t_{R}\quad {\dots }\quad \end{aligned}$$

where ${\bar{\psi }}=\psi ^{\dagger }\gamma ^{0}$ and we have switched to the Dirac representation, i.e.

38 $$\begin{aligned} \gamma ^{0}=\begin{pmatrix} 1&{}0&{}0&{}0\\ 0&{}1&{}0&{}0\\ 0&{}0&{}-1&{}0\\ 0&{}0&{}0&{}-1 \end{pmatrix} \end{aligned}$$

This is identical to the Peierls metal-insulator transition, where the system becomes unstable to to a potential with wavelength $2{\mathbf{k}}_{F}$^31^.

Equivalently, we can take $u_{L}$ and $t_{R}$, and grouping them together gives the Hamiltonian:

39 $$\begin{aligned} \psi ^{\dagger }{\hat{H}}\psi = \begin{pmatrix} u^{\dagger }_{R}\quad t^{\dagger }_{L} \end{pmatrix} \, \begin{pmatrix} \epsilon _{-{\mathbf{k}}}&{}\langle W^{3}_{3}\rangle \\ \langle W^{3}_{3}\rangle &{}\epsilon _{{\mathbf{k}}} \end{pmatrix} \, \begin{pmatrix} u_{R}\\ t_{L} \end{pmatrix} \end{aligned}$$

Diagonalizing gives as eigenvectors the linear combinations:

40 $$\begin{aligned} |{\psi}_{+}\rangle = |u_{R}\rangle + |t_{L}\rangle ,\quad {\text{and}}\quad |{\psi}_{-}\rangle = |u_{R}\rangle - |t_{L}\rangle \end{aligned}$$

with eigenvalues $E_{+} = \epsilon _{{\mathbf{k}}}+\langle W^{3}_{3}\rangle$ and $E_{-} = \epsilon _{{\mathbf{k}}}-\langle W^{3}_{3}\rangle$ assuming that $\epsilon _{{\mathbf{k}}}=\epsilon _{-{\mathbf{k}}}$, i.e. both states sit on the Fermi surface. Time evolving, and computing the probability of transitioning from $t_{L}$ to $u_{R}$ as Rabi oscillations gives:

41 $$\begin{aligned} P_{L\rightarrow R}(t) = {\text{sin}}^{2}\bigg ({\frac{(E_{+}-E_{-})}{2\hbar }}t\bigg ) \end{aligned}$$

Thus the probability of an electron being in either a left- or right-handed state is oscillatory in time, with a frequency given by the magnitude of the phonon VEV: $(E_{+}-E_{-}) = \langle W^{3}_{3}\rangle$. This is precisely the same statement as the “mass” terms in Eq. (37) above, generated from the phonon VEV taking left-handed particles into right-handed and vice versa.

**Figure 4:**
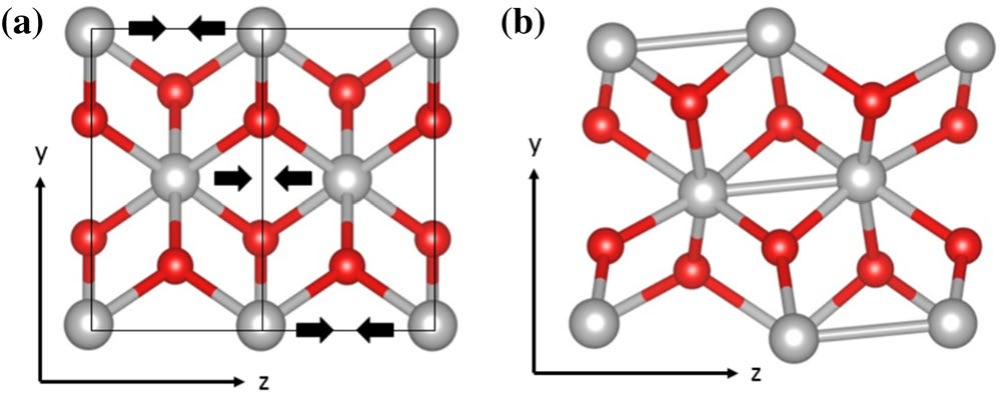
(**a**) Tetragonal structure of ${\text{VO}}_{{2}}$ with the z-axis oriented down the crystallographic c-axis showing the pairing distortion of the MIT, unit cell boundaries are marked in black and (**b**) view of the ${\text{M}}_{{1}} \, {\text{VO}}_{{2}}$ structure showing the Peierls pairs and their relative positions on different vanadium chains. Image produced using VESTA^24^ 3.1.5 (https://jp-minerals.org/vesta/en/).

To give this some context, the aforementioned metal-insulator transition of ${\text{M}}_{{1}} \, {\text{VO}}_{{2}}$ contains just such a Peierls pairing component. Figure 4 illustrates the polarization vectors of the pairing distortion, and as vanadium atoms from neighbouring unit cells are moving towards each other, this defines a zone edge mode, however the pairing displacements also have a non-zero component in the y-direction; the vanadium atoms are moving in opposite directions in neighbouring unit cells along the y-axis, and thus the wavevector of this phonon mode has two non-zero components, ${\mathbf{k}}_{y}$ and ${\mathbf{k}}_{z}$. In vanadium dioxide all three phonon modes acquire VEVs at ${\text{T}}_{{c}}$, and the combination of spin- and charge-ordering in the metal-insulator transition of ${\text{VO}}_{{2}}$ requires a more comprehensive treatment based on an SU(2) *lattice* gauge theory.

There is also the option of breaking the symmetry with the ${\hat{W}}^{\pm }_{\mu }$, however some consideration reveals that giving these phonons a VEV does not result in a ground state with fluctuating spins (a charge VEV will of course exist as the transverse phonons do move the metal atoms). From Fig. 3, and using the $u_{R}$ spinor as an example we see that the ${\hat{W}}^{+}_{\mu }$ can scatter: $u_{R}\rightarrow d_{R}$ (which is not a Dirac mass), or $u_{R}\rightarrow b_{L}$. However, for both processes, giving ${\hat{W}}^{+}_{\mu }$ a VEV will decouple it from the electron spin. Reiterating:

42 $$\begin{aligned} {\hat{W}}^{a}_{\mu }(x)\sim \sum _{p}{\hat{a}}^{\dagger }_{p}\epsilon _{\mu }(p)e^{-ipx} + H.c. \end{aligned}$$

this oscillating polarization vector creates a positive current density $J^{p,\mu } = (\rho ,{\mathbf{J}})$, however the time derivative, or the energy $\hbar \omega$ goes to zero as the phonon gains a VEV. Therefore the time-dependence of the phonon vanishes, and therefore so does the current, and thus the associated magnetic field. Thus, spin ordering is a dynamic process which will occur before the phonon VEV sets in, i.e. above ${\text{T}}_{{c}}$, and below ${\text{T}}_{{c}}$ oscillations of the type described by Eq. (37) which either flip helicity by flipping the spin, or preserve helicity by flipping the spin and the momentum, will not be present in the ground state. Of course spin fluctuations due to the ${\hat{W}}^{\pm }_{\mu \nu }$ propagators can still manifest as excitations, and a charged boson VEV will still couple to the electron charge (i.e. the U(1) gauge charge), but not the SU(2) gauge charge. Thus the onset of a static structure transformation (the phonon VEVs) will cease to dynamically order spins and therefore this ordering must occur above the ${\text{T}}_{{c}}$ of any structural transformation.

#### Lattices, gauge boson mass and the Ward-Takahashi identity

In high energy physics the Ward-Takahashi identity reflects the unphysical nature of the gauge redundancy, and reveals that the longitudinal components of massless vector bosons decouple from scattering amplitudes^32^. By defining a discrete version of the Ward-Takahashi identity, we can see how the symmetry-breaking of Eq. (37) reflects the emergence of a massive boson.

The propagator for a massive transverse spin 1 boson in the Unitary gauge is given by:

43 $$\begin{aligned} W^{a}_{\mu \nu } = \frac{i}{k^{2}-m^{2}+i\epsilon } \bigg (g_{\mu \nu }-\frac{k_{\mu }k_{\nu }}{m^{2}}\bigg ) \end{aligned}$$

For a massless phonon in a crystal with a specific orientation, we now want to express that the polarization vectors are 3-vectors explicitly:

44 $$\begin{aligned} W^{a}_{ij} = \frac{i}{k^{2}+i\epsilon } \bigg (\delta _{ij}- \frac{k_{i}k_{j}}{|{\mathbf{k}}^{2}|}\bigg ) \end{aligned}$$

where ${\mathbf{k}}$ is the 3-momentum, and *k* is the 4-momentum (i.e. ($\epsilon _{{\mathbf{k}}}$, $v_{a}{\mathbf{k}}$)), where *a* labels the boson, and the indices *i*, *j* run from 1 $\rightarrow$ 3, as the polarization vectors have no time component due to the specific orientation of the crystal. We have also assumed linear dispersion for the sake of simplicity, and thus the poles are given by $k^{2}$. The term:

45 $$\begin{aligned} \frac{k_{i}k_{j}}{|{\mathbf{k}}^{2}|} \end{aligned}$$

is the longitudinal component, and thus the longitudinal phonon propagator is

46 $$\begin{aligned} W^{a}_{ij} = \frac{i}{k^{2}+i\epsilon } \bigg (\frac{k_{i}k_{j}}{|{\mathbf{k}}^{2}|}\bigg ) \end{aligned}$$

In continuous systems the scattering term containing this component is zero, which is a trivial manifestation of the Ward-Takahashi identity, while for discrete systems we can see that this will occur for boson momenta which coincide with the reciprocal lattice vectors.

Approximating the full scattering vertex $-igW^{a}_{ij}{\bar{\psi }}\gamma ^{j}\psi$ with $\sim -ig{k}_{j}{\bar{\psi }}\gamma ^{j}\psi$ to see the effect most clearly gives:

47 $$\begin{aligned} \frac{i}{(\gamma ^{\mu }p_{\mu }+\gamma ^{j}k_{j})+i\epsilon } (-ig\gamma ^{j}k_{j})\frac{i}{\gamma ^{\mu }p_{\mu }+i\epsilon } =ig(\gamma ^{j}p_{j}+\gamma ^{j}k_{j}-\gamma ^{j}p_{j}) \bigg (\frac{1}{(\gamma ^{\mu }p_{\mu }+\gamma ^{j}k_{j}) \gamma ^{\mu }p_{\mu }+i\epsilon }\bigg ) \end{aligned}$$

where we have used $\gamma ^{j}k_{j}=(\gamma ^{j}p_{j}+\gamma ^{j}k_{j})-\gamma ^{j}p_{j}$ from momentum conservation at the vertex. It can be seen that the prefactor in parentheses will in general be non-zero, except for the case of a reciprocal lattice vector, ${\mathbf{G}}$ by writing this in operator form acting on the plane wave parts of the tight-binding momentum states:

48 $$\begin{aligned} \gamma ^{j}(p_{j}+G_{j}-p_{j})= & {} -i\gamma ^{j} (\partial _{j}e^{i(p_{j}+G_{j})R_{j}}-\partial _{j}e^{ip_{j}R_{j}}) =-i\gamma ^{j}(\partial _{j}(e^{ip_{j}R_{j}}e^{iG_{j}R_{j}})- \partial _{j}e^{ip_{j}R_{j}}) \\= & {} -i\gamma ^{j}(\partial _{j} e^{ip_{j}R_{j}}-\partial _{j}e^{ip_{j}R_{j}})=0 \end{aligned}$$

where the derivative is taken with respect to the unit cell of the crystal, and we have assumed that the local basis functions of the tight-binding wavefunctions are identical. That is, since

49 $$\begin{aligned} {\psi}_{{\mathbf{k}}}({\mathbf{r}})= \sum _{{\mathbf{R}}}\phi ({\mathbf{r}}-{\mathbf{R}})e^{i\mathbf{kR}} \end{aligned}$$

for a large crystal the sum over lattice vectors will result in the ${\mathbf{R}}$ dependence of the basis functions dropping out, and the only contributions to derivatives taken with respect to ${\mathbf{R}}$ will come from the plane wave term. Thus the local basis function prefactors cancel.

However, if we consider the case of the symmetry-breaking represented by the Hamiltonian of Eq. (39), the diagonalization process gives two states which correspond to the same wavevector. Thus there is now a band index associated with the electronic states $|{\psi}_{+}\rangle$ and $|{\psi}_{-}\rangle$: we have $|{\psi}_{n{\mathbf{p}}}\rangle$, where the index *n* denotes which eigenfunction we are considering. Thus after symmetry-breaking $|{\psi}_{n{\mathbf{p}}}\rangle \ne |{\psi}_{n^{\prime }{\mathbf{p}}}\rangle$, and therefore the identity of Eq. (48) is *not* satisfied for inter-band scattering (i.e. $n\rightarrow n^{\prime }$). While the momentum conservation part of the interaction (Eq. (48)), the basis functions no longer cancel. For example, take pairing of two single basis functions by diagonalization:

50 $$\begin{aligned} {\psi}_{n{\mathbf{p}}}({\mathbf{r}}) = \sum _{{\mathbf{R}}}(\phi _{1}({\mathbf{r}}-{\mathbf{R}})+ \phi _{2}({\mathbf{r}}-{\mathbf{R}}))e^{i{\mathbf{pR}}}, \quad {\psi}_{n^{\prime }{{\mathbf{p}}}}({\mathbf{r}})= \sum _{{\mathbf{R}}}(\phi _{1}({\mathbf{r}}-{\mathbf{R}})- \phi _{2}({\mathbf{r}}-{\mathbf{R}}))e^{i{\mathbf{pR}}} \end{aligned}$$

We see that upon plugging these functions into Eq. (48), the prefactor becomes:

51 $$\begin{aligned}&\gamma ^{j}p_{j}({\psi}_{n{\mathbf{p}}}-{\psi}_{n^{\prime }{\mathbf{p}}}) \\&\quad = -i\gamma ^{j}\big (\partial _{j}\sum _{{\mathbf{R}}}(\phi _{1} ({\mathbf{r}}-{\mathbf{R}})+\phi _{2}({\mathbf{r}}-{\mathbf{R}})) e^{ip_{j}R_{j}}-\partial _{j}\sum _{{\mathbf{R}}}(\phi _{1} ({\mathbf{r}}-{\mathbf{R}})-\phi _{2}({\mathbf{r}}-{\mathbf{R}})) e^{ip_{j}R_{j}}\big )\ne 0 \end{aligned}$$

assuming of course that for a large crystal the ${\mathbf{R}}$-dependence of the basis functions disappears due to the sum over ${\mathbf{R}}$, although this will not be true near the boundary of the crystal (the integration by parts to produce the propagator has a boundary term which will contribute in this region). Therefore the longitudinal component of the propagator does not vanish for scattering by reciprocal lattice vectors, there is a new pole which is not at $k^{\mu }k_{\mu }$, the zero component of the 4-momentum has an extra energy term corresponding to $\epsilon _{n{\mathbf{p+G}}}-\epsilon _{n^{\prime }{\mathbf{p}}}$: the boson has acquired a mass. This component *does* still vanish for intra-band transitions (i.e. $n = n^{\prime }$), and therefore there are both massive and massless phonons: the optical and acoustic branches. Thus similarly to particle physics, the violation of the Ward-Takahashi identity signifies the presence of massive bossons, in this case: optical phonons.

## Conclusion

Postulating that the observation of spin and charge ordering appearing coincident with different crystal structure distortions allows an SU(2) Yang-Mills theory of electron-phonon interactions in vanadium dioxide to be formulated. Due to this resolution it is possible to use the electron spin and charge/crystal momentum to group the spinors into forms which when acted on by the phonons produces both spin- and charge-ordering observed in crystal structure transformation such as those of the polymorphs of vanadium dioxide.

The interaction vertex in this case straddles neighbouring vanadium metal ions. That is, it acts simultaneously on pairs of cations, which breaks the symmetry and results in charge and spin-structure transformations. This effectively “links” neighbouring metal ions, which given the strong electron correlations is to be expected: it is likely that any persistent structural distortion must be lowering electron correlations. An example of this is the antiferroelectric distortion of ${\text{M}}_{{2}} \, {\text{VO}}_{{2}}$ discussed above, this distortion lowers overlap between neighbouring wavefunctions as the metal ions move further apart, reducing bandwidth and the probabilities of electron hopping and thus double occupancies.

While such an interaction vertex is more complicated than the usual formalism such as that of Bardeen and Pines^33^, its complexity is a strength as it explicitly contains useful interactions due to being built on effective interactions between phonon polarization vectors and spinor variables. It is also formalism that has a long history of study and simulation using Monte Carlo methods by high energy physicists, such as in the field of Quantum Chromodynamics^34^. The natural extension for this work is to adapt lattice gauge simulation methods to the study of electron-phonon interactions in strongly correlated systems.

## Methods

The GW calculations were performed using the implementation of Shishkin and Kresse^35,36^ as contained in the Vienna Ab Initio Simulation Package (VASP)^37^, after first calculating input wavefunctions using DFT^38^ with GGA^39^ functionals, on $8\times 8\times 6$ and $4\times 6\times 6$ Monkhorst-Pack^40^ **k**-space grids for the Tetragonal and ${\text{M}}_{{2}}$ structures respectively, using the Brillouin zone integration approach of Bloechl et al.^41^.
